# Chemical shift assignments of calmodulin bound to the β-subunit of a retinal cyclic nucleotide-gated channel (CNGB1)

**DOI:** 10.1007/s12104-022-10072-9

**Published:** 2022-02-02

**Authors:** Aritra Bej, James B. Ames

**Affiliations:** Department of Chemistry, University of California, Davis, CA 95616 USA

**Keywords:** CaM, Calcium, CNGB1, Retina, Photoreceptor, NMR

## Abstract

Rod cyclic nucleotide-gated (CNG) channels are formed by two protein subunits (CNGA1 and CNGB1). Calmodulin (CaM) binds to the cytosolic regulatory domain of CNGB1 and decreases the open probability of CNGA1/CNGB1 channels. The CaM binding site within bovine CNGB1 (residues 679–702) binds tightly to Ca^2+^-bound CaM, which promotes Ca^2+^-induced inactivation of CNGA1/CNGB1 channels in retinal rods. We report complete NMR chemical shift assignments of Ca^2+^-saturated CaM bound to the CaM-binding domain of CNGB1 (BMRB no. 51222).

## Biological context

Cyclic nucleotide-gated (CNG) channels expressed in retinal rods conduct a cation current in response to changes in intracellular levels of cGMP that occur during visual phototransduction (Baylor 1996, Fesenko, Kolesnikov et al. 1985). Ca^2+^-dependent regulation of photoreceptor CNG channels by CaM is important for promoting light adaptation in photoreceptor cells (Bradley et al. 2005, Fain et al. 2001, Hsu and Molday 1993). Retinal CNG channels consist of two protein subunits, CNGA1 and CNGB1 (Bradley et al. 2001). The CNGA1 subunit can form a functional homotetrameric channel when expressed alone, whereas CNGB1 does not form a functional homomeric channel (Finn et al. 1998). Native CNG channels in retinal rods form a heteromeric tetramer comprised of a 3:1 stoichiometry of CNGA1:CNGB1 (Shuart, Haitin et al., 2011). Three CNGA1 subunits form a trimer that binds tightly with a single CNGB1 subunit in a Ca^2+^-dependent fashion. The Ca^2+^ sensor protein, calmodulin (CaM) binds to a cytosolic site in CNGB1 (residues 679-702) (Trudeau and Zagotta 2002) that may regulate CNGB1 binding to CNGA1 (Shuart et al. 2011) and perhaps mediate Ca^2+^-induced CNG channel inactivation in rod cells (Hsu and Molday 1993; Trudeau and Zagotta 2003). Defects in the Ca^2+^-dependent regulation of CNG channels are genetically linked to autosomal recessive retinitis pigmentosa and other inherited forms of blindness (Bareil, Hamel et al. 2001). Elucidating the CNG channel structural interaction with CaM may provide insights for the treatment of retinal diseases.

Although structures are known for CaM bound to the CNGA2 subunit from olfactory CNG channels (Contessa et al. 2005), atomic level structural information is currently not known for CaM bound to the retinal CNGB1. We report here NMR resonance assignments of Ca^2+^-saturated CaM bound to the CaM-binding domain of CNGB1 (hereafter called CaM/CNGB1). These assignments are a first step toward elucidating the structure of CaM bound to CNGB1.

## Methods and experiments

### Expression and purification of CaM

Recombinant human CaM was subcloned into pET11b expression vector (Novagen) and overexpressed in E. coli strain BL21(DE3) as described previously (Turner, Anderson et al. 2020). Uniformly ^13^C/^15^N-labeled CaM samples were overexpressed in M9 minimal media, containing 1 g/L ^15^NH_4_Cl and 3 g/L ^13^ C-labeled glucose (Cambridge Isotopes Laboratories) as the sole nitrogen and carbon sources, respectively. The soluble fraction of the cell lysate was loaded onto a HiPrep Phenyl Sepharose 6 column that was pre-equilibrated with equilibration buffer, containing 20 mM Tris (pH 7.5), 200 mM KCl, 2 mM CaCl_2_. The CaM protein was eluted from the column using a buffer that contained 20 mM Tris (pH 7.5), 50 mM KCl, 2 mM EGTA. The eluted protein fraction was further loaded onto a HiPrep Q Sepharose anion exchange column that was pre-equilibrated with 50 mM Tris (pH 7.5), 25 mM KCl, 1 mM EGTA and eluted by a KCl gradient up to 625 mM. The purity and identity of the eluted protein fractions were confirmed by sodium dodecyl sulfate-polyacrylamide gel electrophoresis. A peptide fragment of the CaM binding domain from CNGB1 (residues 679–702) was purchased from GenScript, dissolved in DMSO-d_6_, and quantified using UV–Vis absorption spectroscopy. A 1.7-fold excess of the peptide was added to Ca^2+^-bound CaM, incubated at room temperature for 30 min, and concentrated to 0.5 mM.

### NMR spectroscopy

**Fig. 1:**
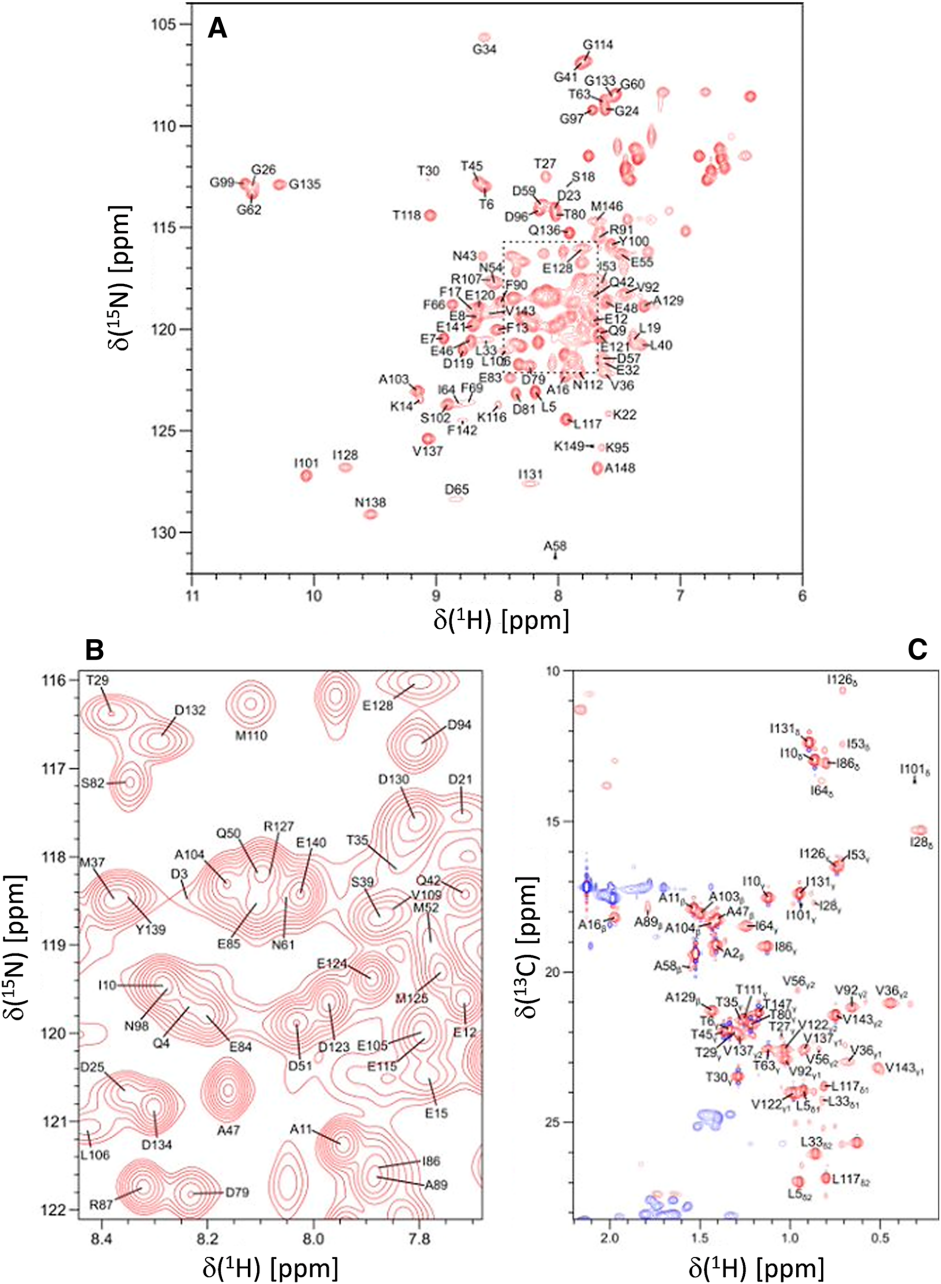
Two-dimensional NMR spectra of CaM bound to unlabeled CNGB1-CaMBD peptide. **A** ^15^N–^1^ H HSQC spectrum recorded at 600 MHz ^1^H frequency was analyzed to determine backbone resonance assignments. **B** Expanded view of resonance assignments from the spectrally crowded region highlighted by the dashed box. **C** Constant-time ^13^C–^1^H HSQC spectrum was analyzed to determine side chain resonance assignments. Representative resonance assignments are indicated by residue labels; complete assignments are available as BMRB accession no. 51222

Protein samples of ^15^ N- or ^13^ C/^15^ N-labeled CaM bound to unlabeled CNGB1 peptide were exchanged into NMR buffer containing 20 mM Tris-d_11_ (pH 7.0) with 1 mM CaCl_2_, and 92% H_2_O/8% D_2_O. The CaM/CNGB1 complex was concentrated to give a final concentration of 0.5 mM in a final volume of 0.3 mL. All NMR experiments were performed at 308 K on a Bruker Avance III 600 MHz spectrometer equipped with a four-channel interface and triple resonance cryogenic (TCI) probe. The ^15^ N–^1^H HSQC spectrum (Fig. 1A, B) was recorded with 256 × 2048 complex points for ^15^N(F1) and ^1^H(F2). Assignment of backbone resonances was obtained by analyzing the following spectra: HNCACB, CBCA(CO)NH, HNCO and HBHA(CO)NH. The assignment of side chain (aliphatic (Fig. 1C) and aromatic) resonances was obtained by analyzing the following spectra: HCCCONH-TOCSY, HCCH-TOCSY, HBCBCGCDHD and HBCBCGCDCEHE as described previously (Ikura et al. 1991). The NMR data were processed using NMRPipe and analyzed using Sparky.

## Extent of assignments and data deposition

**Fig. 2:**
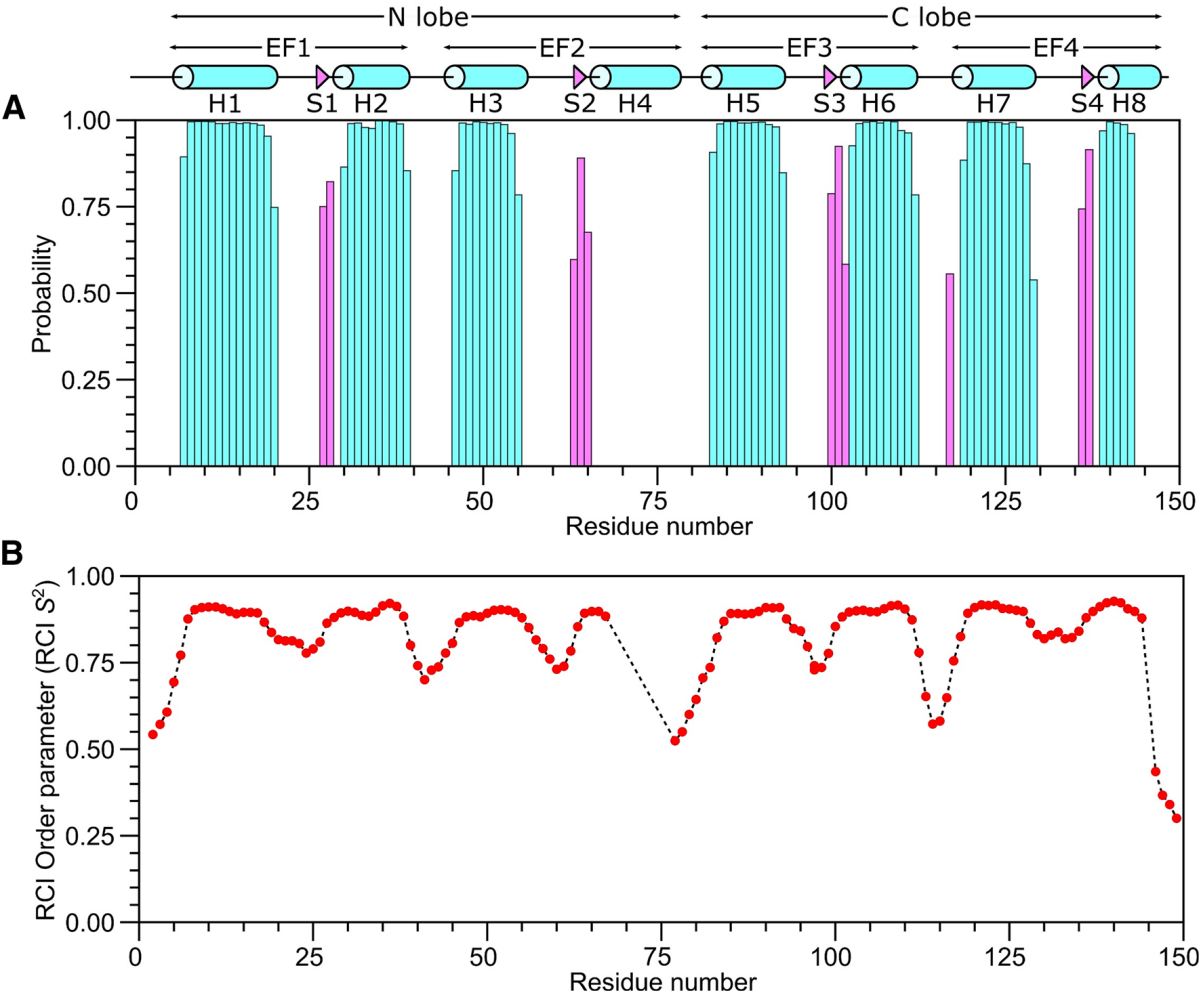
Secondary structure and order parameters of Ca^2+^-saturated CaM bound to unlabeled CNGB1 peptide predicted from the assigned backbone chemical shifts. **A** Probability of secondary structural elements (cyan for helix and magenta for strand) and **B** RCI order parameter (RCI-S^2^) of Ca^2+^-saturated CaM bound to unlabeled CNGB1 peptide were predicted using TALOS+ server (Shen et al. 2009). The wire diagram depicting the secondary structural elements (cylinder for helix and triangle for strand) was obtained from the CaM structure [PDB ID—2VAY (Halling et al. 2009)]

Figure 1A, B present the ^15^N–^1^H HSQC spectrum of CaM/CNGB1 to illustrate representative backbone resonance assignments. Figure 1C presents a constant-time ^13^C–^1^H HSQC spectrum to illustrate side chain methyl resonance assignments. The NMR assignments were based on 3D heteronuclear NMR experiments performed on ^13^C/^15^N-labeled CaM bound to unlabeled CNGB1 peptide. The NMR spectra of CaM/CNGB1 exhibited well-dispersed peaks indicative of a stably folded structure. Four amide resonances (assigned to G26, G62, G99 and G135) exhibited noteworthy downfield shifts that are caused by Ca^2+^ binding to each of the four EF-hands (Fig. 1A). Ring current shifted methyl resonances assigned to residues I28, V36, and I101 (Fig. 1C) suggest these methyl groups are near aromatic residues in the hydrophobic core. More than 85% of the backbone resonances (^1^HN, ^15^ N, ^13^Cα, ^13^Cβ, and ^13^CO) and 83% of aliphatic and aromatic side-chain resonances were assigned. A stretch of ten residues (residues 68–78) in the second EF-hand of CaM could not be assigned (central gap near H4 in Fig. 2), because their HSQC peaks were either broadened beyond detection or otherwise could not be detected. The observed peak broadening here suggests that these residues might undergo conformational exchange processes perhaps caused by their interaction with the bound peptide. Indeed, these same resonances are exchange broadened in CaM bound to the α-subunit of the retinal cyclic nucleotide-gated channel (CNGA2) (Contessa et al. 2005), but are not exchange broadened in free CaM (Kainosho et al. 2006). The chemical shift assignments (^1^H, ^15^N, ^13^C) for CaM/CNGB1 have been deposited in the BioMagResBank (http://www.bmrb.wisc.edu) under accession number 51222.

**Fig. 3:**
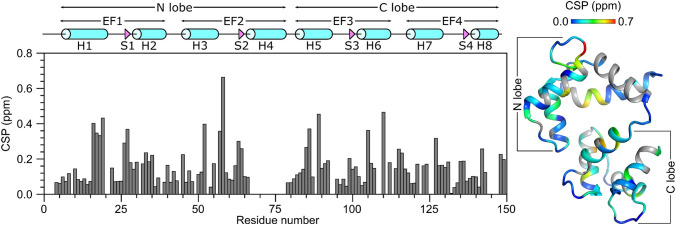
Residue-specific amide chemical shift perturbation (CSP) for Ca^2+^-bound CaM in the presence and absence of CNGB1 peptide. CSP was calculated as: $CSP= \sqrt{{\left(\Delta{H}^{N}\right)}^{2}+{\left(\Delta{N}\right)}^{2}}$. ΔH^N^ and ΔN are the observed difference in the ^1^H^N^ and ^15^N chemical shifts, respectively for CaM/CNGB1 compared to CaM alone. CSP values are mapped on to the CaM structure (PDB ID: 2VAY (Halling et al. 2009))

The secondary structure of CaM/CNGB1 was calculated based on the chemical shift index (Wishart et al. 1992) of each assigned amino acid residue and ANN-Secondary structure prediction using TALOS+ (Shen et al. 2009) (Fig. 2). CaM/CNGB1 contains the following α-helices: H1 (residues 7–20), H2 (residues 30–39), H3 (residues 46–55), H5 (residues 83–93), H6 (residues 103–112), H7 (residues 119–129) and H8 (residues 139–143) depicted by cylinders in Fig. 2A. Four short β-strands named S1 (residues 27–28), S2 (residues 63–65), S3 (residues 100–102) and S4 (residues 136–137) are depicted by the triangles in Fig. 2A. Preliminary NMR-derived distance restraints inferred from NOESY spectra suggest that the observed α-helices and β-strands combine to form 4 EF-hand Ca^2+^ binding motifs (EF1: residues 7–39, EF2: residues 45–76, EF3: residues 83–112 and EF4: residues 119–144) as seen in the crystal structure of CaM in the absence of peptide (Babu et al. 1988). In the CaM crystal structure, the N-terminal EF-hands (EF1 and EF2) interact to form what is called the N-lobe, while EF3 and EF4 interact to form the C-lobe. The binding of the CNGB1 peptide to CaM causes detectable chemical shift perturbations that are distributed uniformly throughout both the N-lobe and C-lobe of CaM (Fig. 3). Thus, the CNGB1 peptide is likely making contact with both lobes of CaM, consistent with the familiar collapsed structure of CaM bound to other peptide targets (Hoeflich and Ikura 2002). The CaM residues (A16, L19, L33, M52, A89, L106, M110 and F142) that have relatively high CSP values in Fig. 3 correspond to the residues that directly contact the CNGA2 peptide in the NMR structure of CaM/CNGA2 (Contessa et al. 2005). The NMR assignments of CaM/CNGB1 presented here are an important first step toward determining its full three-dimensional structure.

## Data Availability

The assignments have been deposited to the BMRB under the accession code: 51222.
